# CoA synthase plays a critical role in neurodevelopment and neurodegeneration

**DOI:** 10.3389/fncel.2024.1458475

**Published:** 2024-09-05

**Authors:** Chiara Cavestro, Marco D’Amato, Maria Nicol Colombo, Floriana Cascone, Andrea Stefano Moro, Sonia Levi, Valeria Tiranti, Ivano Di Meo

**Affiliations:** ^1^Unit of Medical Genetics and Neurogenetics, Fondazione IRCCS Istituto Neurologico Carlo Besta, Milan, Italy; ^2^Vita-Salute San Raffaele University, Milan, Italy; ^3^Division of Neuroscience, IRCCS San Raffaele Scientific Institute, Milan, Italy

**Keywords:** CoA synthase, COASY protein-associated neurodegeneration, pontocerebellar hypoplasia type 12, neurodegeneration, neurodevelopment, animal models

## Abstract

Coenzyme A (CoA), which is widely distributed and vital for cellular metabolism, is a critical molecule essential in both synthesizing and breaking down key energy sources in the body. Inborn errors of metabolism in the cellular *de novo* biosynthetic pathway of CoA have been linked to human genetic disorders, emphasizing the importance of this pathway. The *COASY* gene encodes the bifunctional enzyme CoA synthase, which catalyzes the last two reactions of the CoA biosynthetic pathway and serves as one of the rate-limiting components of the pathway. Recessive variants of this gene cause an exceptionally rare and devastating disease called COASY protein-associated neurodegeneration (CoPAN) while complete loss-of-function variants in *COASY* have been identified in fetuses/neonates with Pontocerebellar Hypoplasia type 12 (PCH 12). Understanding why the different symptoms emerge in these disorders and what determines the development of one syndrome over the other is still not achieved. To shed light on the pathogenesis, we generated a new conditional animal model in which *Coasy* was deleted under the control of the human GFAP promoter. We used this mouse model to investigate how defects in the CoA biosynthetic pathway affect brain development. This model showed a broad spectrum of severity of the *in vivo* phenotype, ranging from very short survival (less than 2 weeks) to normal life expectancy in some animals. Surviving mice displayed a behavioral phenotype with sensorimotor defects. *Ex vivo* histological analysis revealed variable but consistent cerebral and cerebellar cortical hypoplasia, in parallel with a broad astrocytic hyper-proliferation in the cerebral cortex. In addition, primary astrocytes derived from this model exhibited lipid peroxidation, iron dyshomeostasis, and impaired mitochondrial respiration. Notably, *Coasy* ablation in radial glia and astrocytic lineage triggers abnormal neuronal development and chronic neuroinflammation, offering new insights into disease mechanisms.

## Introduction

Coenzyme A (CoA) is an essential cofactor that plays a central role in maintaining cellular homeostasis. It is involved in fundamental processes such as cell growth and death, energy production, protein modification, and regulation of gene expression (Srinivasan and Sibon, 2014). The biosynthesis of CoA occurs via a highly conserved *de novo* metabolic mechanism. The crucial function of CoA in maintaining homeostasis has been confirmed by the discovery of mutations linked to human disorders in all four genes encoding the enzymes involved in CoA biosynthesis (Cavestro et al., 2023).

The *de novo* biosynthetic pathway of CoA consists of five highly conserved reactions, with pantothenate (vitamin B5) serving as the substrate (Robishaw and Neely, 1985). The initial step is the phosphorylation of pantothenate to 4′-phosphopantothenate (PPan), catalyzed by pantothenate kinase (PANK). There are four isoforms of the PANK enzyme (PANK1-4), but only PANK1-3 are involved in kinase activity. The second step involves the formation of 4′-phospho-N-pantothenoylcysteine (PPan-Cys) by Phosphopantothenoylcysteine synthetase (PPCS), combining PPan and cysteine. Phosphoenoylcysteine decarboxylase (PPCDC) catalyzes the subsequent decarboxylation process, resulting in 4′-phosphopantetheine (PPanSH). The last two steps are catalyzed by the bi-functional enzyme CoA synthase (COASY), which consists of two catalytic domains: phosphopantetheine adenylyl-transferase (PPAT) and dephospho-CoA kinase (DPCK). First, it undergoes an adenylation reaction to form dephospho-CoA (dPCoA), which is then phosphorylated to CoA. COASY is predominantly located in the mitochondrial matrix and on the outer mitochondrial membrane (Zhyvoloup et al., 2003; Rhee et al., 2013), with some reports indicating free cytoplasmic and nuclear localization (Lin et al., 2018).

Pathological variants in the genes encoding these four enzymes are associated with two categories of human diseases. Mutations in *PPCS* and *PPCDC* are associated with forms of dilated cardiomyopathy (Iuso et al., 2018; Lok et al., 2022; Bravo-Alonso et al., 2023), while variants in *PANK2* and *COASY* cause neurological disorders classified as Neurodegenerations with Brain Iron Accumulation (NBIA). Variants in *PANK2* cause pantothenate kinase-associated neurodegeneration (PKAN) (OMIM: #234200) (Zhou et al., 2001), while mutations in *COASY* cause COASY Protein-Associated Neurodegeneration (CoPAN) (OMIM: #615643) (Dusi et al., 2014). Although these disorders vary in frequency, they share many neurological symptoms, including Parkinsonian features, cognitive impairment, obsessive-compulsive symptoms, and MRI-detected brain iron accumulation in the basal ganglia (Cavestro et al., 2023).

Recent studies have highlighted a wide range of symptoms associated with variants in the *COASY* gene. Thirteen fetuses/newborns with complete loss-of-function *COASY* variants exhibited pontocerebellar hypoplasia type 12 (PCH12), a fatal inherited neurodevelopmental disorder characterized by microcephaly and perinatal death, without brain iron accumulation (van Dijk et al., 2018; Mishra et al., 2022). Other patients, with or without brain iron accumulation, presented with varying degrees of neurological symptoms, including speech delay, mild movement defects, dysarthria, dysphagia, hypotonia, atrophy of the brainstem and basal ganglia, developmental delay, and status epilepticus, often in association with microcephaly and facial dysmorphism (Ajit et al., 2023; Hashemi et al., 2023; Mahale et al., 2023; Rosati et al., 2023).

More recently, sixteen individuals with *COASY* variants were found to have riboflavin-responsive lipid storage myopathy (RR-LSM), characterized by exercise intolerance or muscle weakness (Zheng et al., 2024). A recent study published five new patients with four distinct COASY variants, displaying classic or previously unreported clinical characteristics, including seizures, hearing loss, strabismus, autism spectrum disorder, and neuromuscular involvement with multiple mitochondrial complex defects in muscle (Cavestro et al., 2024). Despite the broad spectrum of conditions, it remains unclear how distinct symptoms arise and what factors determine the development of one condition over another, such as neurodevelopment impairment or neurodegeneration.

After the first CoPAN patients were identified, several studies were carried out to clarify some aspects of the pathology using model organisms. The *Saccharomyces cerevisiae* model, in which the two enzymes that fulfill the catalytic function of Coasy have been knocked out, is the simplest model developed to date. This leads to a lethal effect and the re-expression of a human-mutant COASY protein produces a reduction in mitochondrial CoA, mitochondrial dysfunction, and iron accumulation (Olzhausen et al., 2013; Ceccatelli Berti et al., 2015). In zebrafish, complete abrogation of *COASY* expression caused high mortality and abnormal embryonic development, while partial reduction led to almost normal development with poorly defined brain structure and head edema. This study gives the first evidence of the fundamental role of *COASY* in brain development (Khatri et al., 2016). Recently, we found that constitutive ablation of *Coasy* in mice is not compatible with life, while conditional, neuronal-specific *Coasy* loss showed severe early-onset neurological phenotypes, locomotor abnormalities, dystonia-like movements, growth arrest, and invariable premature death by postnatal day 15. Brain examinations revealed altered mitochondrial structure and function, as well as altered iron homeostasis, but without signs of impaired neurodevelopment, nor post-mitotic neuronal loss (Di Meo et al., 2020).

To further investigate the role of the COASY protein in the brain, here we generated another mouse model in which *Coasy* was conditionally deleted using the Cre transgene under the control of the human glial fibrillary acidic protein (GFAP) promoter, which is active in radial glial cells during the development, and in astrocytes in the adult central nervous system (CNS) (Zhuo et al., 2001; Malatesta et al., 2003). This model (GFAP-Coasy) showed a wide range of severity, from extremely short survival (approximately 2 weeks) to normal life expectancy. Short-surviving animals displayed severe cortical and cerebellar hypoplasia, probably due to radial glia loss or impaired differentiation. Otherwise, long-surviving mice showed sensorimotor abnormalities, defects in motor coordination, and signs of variable but consistent cerebral and cerebellar cortical abnormalities on *ex vivo* histological analysis. Widespread astrogliosis and cerebral iron dyshomeostasis were also observed. Our study provides new insights into the role of COASY during brain development and in the various cerebral cellular components. By specifically removing the protein in neurons (Di Meo et al., 2020) and here in the astroglial lineage, we were able to dissect the contribution of each neuronal type to the manifestation of clinical phenotypes based on the presence or absence of the COASY protein.

## Materials and methods

### Animal studies

Coasy^flox/flox^ mice (Di Meo et al., 2020) were crossed with transgenic animals expressing the Cre transgene under human glial fibrillary acidic protein (hGFAP) promoter (JAX stock no. 004600) to obtain the Coasy^+/flox^, +/GFAP-Cre mice. Further mating cycles allowed to obtain Coasy^flox/flox^, +/GFAP-Cre (GFAP-Coasy) mice. Animals were housed in an animal care facility with controlled temperature (21 ± 2° C) and relative humidity (55 ± 10%), with a 12 h light/dark cycle; water and food were provided *ad libitum*. The mice were maintained in a mixed background of C57Bl6/129Sv. Body weight was registered weekly, while motor coordination and balance were assessed monthly by rotarod and pole tests as previously described (Brooks and Dunnett, 2009). Cervical dislocation was used to perform euthanasia and the brain was quickly excised and treated appropriately for subsequent uses: frozen in liquid nitrogen for molecular analysis, or post-fixed in paraformaldehyde 4% for 24 h at 4°C, cryoprotected in PBS containing 30% sucrose for 48 h at 4°C, and then frozen in isopentane.

### Immunohistochemistry and immunofluorescence

For immunohistochemical procedures, a cryostat (Leica Biosystems, Buccinasco, Italy) was used to cut brain slices (30 μm), which were then preserved at 4°C in PBS with 0.1% sodium azide. Sections were incubated in H_2_O_2_ 0.1% for 10 min, followed by a PBS wash and incubation with the blocking solution (PBS containing 10% FBS and 0.3% Triton X-100). Slices were incubated with various primary antibodies, such as NeuN (MAB377, Millipore, Burlington, MA, United States), GFAP (Z0334, Dako, Japan), Calbindin (EP3478, Abcam, Cambridge, United Kingdom), and then with goat anti-rabbit or anti-mouse biotinylated secondary antibodies (Jackson Immunoresearch, Ely, United Kingodm). Following the manufacturer’s directions, Vectastain Elite ABC kit (Vector, Burlingame, CA, United States) and DAB solution (Vector, Burlingame, CA, United States) were used to detect signals. Subsequently, sections were mounted on gelatin-coated glass slides, dehydrated, cleared in Bioclear (Bio-Optica, Milan, Italy), and covered with DPX (Sigma-Aldrich, Milan, Italy). Under bright-field illumination, labeled structures were inspected using a Zeiss Axioplan 2 microscope (Zeiss, Milan, Italy). Digital images were captured with a Zeiss camera using AxioVision software (Zeiss, Milan, Italy).

For immunofluorescence staining, primary astrocytes were fixed in paraformaldehyde 4% for 15 min, treated with blocking solution, and then incubated with GFAP (Z0334, Dako, Japan) and EAAT2 (sc-365634, Santa Cruz Biotechnology, Dallas, TX, USA) primary antibodies, and with goat anti-rabbit IgG (Alexa Fluor 488-conjugated, Invitrogen, Waltham, MA, USA), goat anti-mouse IgG (Alexa Fluor 568-conjugated, Invitrogen, Waltham, MA, USA) secondary antibodies. Images were acquired on a TCS-SP8 laser confocal microscope using LAS AF software (Leica Biosystems, Buccinasco, Italy).

### Real-time quantitative PCR

Total RNA was extracted using the TRIzol reagent (Life Technology, Waltham, MA, USA), and then purified using the RNeasy mini kit (QIAGEN, Hilden, Germany). Using the GoScript Reverse Transcriptase kit (Promega, Madison, WI, USA), RNA was reverse transcribed into cDNA, and qPCR was performed in duplicate using the SsoAdvanced Universal Probes Supermix (Bio-Rad, Hercules, CA, USA), and the TaqMan™ Gene Expression Assays (Thermo Fisher Scientific, Waltham, MA, USA) for Coasy (Mm01292513_g1) and Actb (Mm02619580_g1). The amount of Coasy cDNA was compared using the ΔΔCq method, relative to the beta-actin (Actb) gene.

### Immunoblotting

Mouse tissues were homogenized in 25 volumes of ice-cold RIPA buffer (Tris–HCl 50 mM pH 7.5, NaCl 150 mM, EDTA 5 mM, NP401%, SDS 0.1%, and sodium deoxycholate 0.5%) with the addition of protease inhibitors (Roche, Basel, Switzerland), using a Dounce homogenizer. Isolated astrocytes were harvested by trypsinization and incubated in 50 to 100 μL of RIPA buffer depending on the cell number. Following a 30-min ice incubation period, homogenates were centrifuged at 18,000 x g for 30 min at 4°C and then the protein content in the soluble fraction was assessed using the DC™ Protein Assay Kit (BioRad, Hercules, CA, United States). A quantity of 10 to 35 μg of total proteins were separated using SDS-PAGE and then electroblotted onto a nitrocellulose membrane, which was subsequently incubated with specific antibodies for COASY (PA5-28696, Thermofisher Scientific, Waltham, MA, United States), DCX (#4604, Cell Signaling, Danvers, MA, United States), GFAP (MAB360, Millipore, Burlington, MA, United States), Ft-L and Ft-H (Santambrogio et al., 2000), GAPDH (ab181602, Abcam, Cambridge, United Kingdom), TfR1 (13–6,800, Thermofisher Scientific, Waltham, MA, United States), DMT1 (sc-166884, Santa Cruz Biotechnology, Dallas, TX, United States), ACO2 (NBP1-32781, Novus Biologicals, Centennial, CO, United States), NCOA4 (sc-373739, Santa Cruz Biotechnology, Dallas, TX, United States). ImageJ software was used for the densitometry quantification.

### Mitochondrial complex I analysis

Tissues were homogenized in 10 mM phosphate buffer (pH 7.4) using a Dounce homogenizer, and the spectrophotometric activity of complex I was measured as previously described (Bugiani et al., 2004).

### Isolation of primary astrocytes

The isolation of astrocytes from the mouse brain was performed as reported (Schulte et al., 2016) with minor modifications. Briefly, the hippocampi of three mice for each genotype (GFAP-Coasy and controls) were harvested on the third day of life. The tissues were cut into pieces and put in Dix-Med (4.17 mM NaHCO_3_, 10 mM HEPES, 6 mg/mL glucose, 200 μM kynurenic acid, 0.3 mg/mL BSA, 12 mM magnesium sulphate, 5 μg/mL gentamycin, in Hank’s salt solution, pH 7.3) and washed twice. After three other washes in Hanks Buffer (10 mM HEPES, 6 mg/mL glucose, 200 uM kynurenic acid, in Hank’s salt solution, pH 7.3), we performed enzymatic digestion with Digestion Buffer (137 mM NaCl, 5 mM KCl, 7 mM NaHPO4, 25 mM HEPES, 4 mM NaHCO3, 200 μM kynurenic acid, 3 mg/mL trypsin, 0.6 mg/mL DNAase I), 5 min at room temperature. After three washes in Dix-Med, the tissue was incubated twice with 5 mL Trypsin-inhibitor (Sigma-Aldrich, Milan, Italy) 1 mg/mL in Dix-Med for 5 min in ice. After three washes in Dix-Med, tissue was mechanically dissociated with a serological pipette in a solution of 0.5 mg/mL DNAse I Dix-Med. The suspension was centrifuged for 10 min at 900 x g at 4°C. The pellet was resuspended in high glucose Dulbecco’s Modified Eagle Medium (Euroclone, Pero, Italy) supplemented with 10% fetal calf serum (Euroclone, Pero, Italy), 4 mM glutamine (Euroclone, Pero, Italy), and 1% penicillin/streptomycin (Euroclone, Pero, Italy), plated on T25 Flasks coated with poly-L-lysine 10 μg/mL, and cultured at 37°C in a 5% CO_2_ humidified atmosphere. Microglial cells were removed by shaking the plate.

### Measurement of membrane lipid peroxidation

Lipids peroxidation was analyzed using BODIPY 581/591 C11 dye (Invitrogen, Waltham, MA, USA), following the manufacturer’s instructions. Briefly, cells were incubated with 5 μM of the probe in HSSB for 30 min at 37°C and confocal images were acquired on a TCS-SP8 laser confocal microscope using LAS AF software (Leica Biosystems, Buccinasco, Italy). Images were analyzed with ImageJ software.

### Determination of respiratory activity

The oxygen consumption rate (OCR) and the extracellular acidification rate (ECAR) in astrocytic cells were determined using an Agilent XF96 Extracellular Flux Analyzer (Agilent, Milan, Italy) as previously described (Zanuttigh et al., 2023). Briefly, cells were seeded in Seahorse 96-well plates at 20,000 cells/well density, and OCR was measured in basal conditions, and after sequentially addition of 1 μM oligomycin, 0.4 μM carbonyl cyanide-4-(trifluoromethoxy) phenylhydrazone (FCCP), and 2 μM/2.5 μM rotenone / antimycin A. The CyQUANT™ Direct Cell Proliferation Assay (Invitrogen, Waltham, MA, USA) was used at the end of the procedure to normalize the obtained OCR and ECAR data to the total number of cells in each well.

### Statistical analysis

Prism 9 (GraphPad, Boston, MA, USA) was used to analyze the data. One- or two-way ANOVA, and the two-tailed unpaired Student’s t-test were used, along with the Bonferroni post-hoc test. The mean ± SD is given for the data. *p*-values less than 0.05 were deemed statistically significant.

## Results

### Phenotyping of GFAP-Coasy mouse model

**Figure 1:**
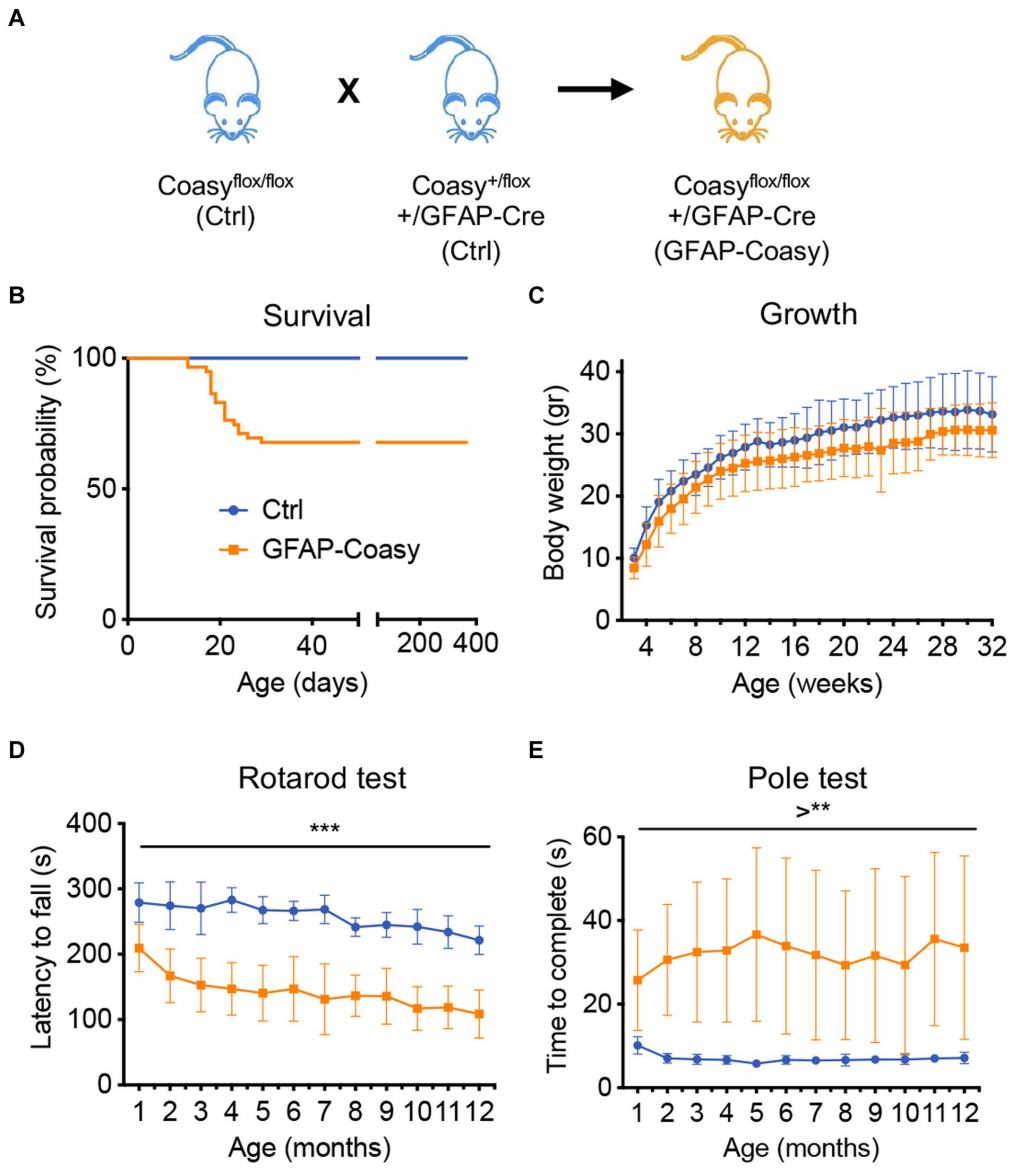
Phenotype characterization of GFAP-Coasy mice. **(A)** Breeding scheme to generate GFAP-Coasy mice. **(B)** Kaplan–Meier survival probability in GFAP-Coasy (*n* = 30) mice. Note that a group of animals dies around 15–25 postnatal days (EO GFAP-Coasy), while the remaining mice are still alive after 400 days (LO GFAP-Coasy). **(C)** Body weights variation, **(D)** rotarod test, and **(E)** pole test analyses in Ctrl (*n* = 15) and LO GFAP-Coasy (*n* = 15). **p < 0.01; ***p < 0.001 (multiple unpaired *t*-test).

Previously generated floxed mice, in which the region encompassing exons two to nine of the *Coasy* gene is flanked by loxP sites (Coasy^flox^) (Di Meo et al., 2020), were crossed with a transgenic mouse expressing the Cre recombinase under the control of the human GFAP promoter (Zhuo et al., 2001) that is active in the developing CNS starting at embryonic day E13 in radial glia and then in adult astrocytes. These mice, Coasy^flox/flox^;hGFAP-Cre, henceforth called GFAP-Coasy (Figure 1A), were obtained respecting a Mendelian transmission of the trait. However, after birth, GFAP-Coasy mice exhibited two distinctly different clinical phenotypes: one characterized by a severe early-onset (EO) pathological condition requiring compassionate euthanasia between postnatal days P15 and P20, and one characterized by a late-onset (LO) clinical phenotype (Figure 1B) that did not affect survival. EO GFAP-Coasy mice showed neurological signs characterized by splayed legs, postural instability leading to loss of balance and falling over, involuntary turning movements, slowness in movement, and awkwardness at righting themselves (Supplementary Video S1). The growth curve of the LO GFAP-Coasy mice was not significantly different from that of control mice, although GFAP-Coasy mice had lower body weight (Figure 1C). At 1 month of age, LO GFAP-Coasy mice showed movement disorders characterized by impaired balance and coordination as measured by the rotarod test (Figure 1D) and this movement disorder worsened as the mice aged (from month 1 to month 12). To further assess motor dysfunction, we measured the time required to turn fully downward and reach the ground by performing the pole test. The time required for LO GFAP-Cosay mice to perform the test was significantly increased compared to controls, and this time continued to increase during the observation period (from month 1 to month 12) (Figure 1E). We could not detect any differences in phenotype manifestations between male and female EO or LO GFAP-Coasy animals.

### Pathological characterization of GFAP-Coasy mouse model

**Figure 2:**
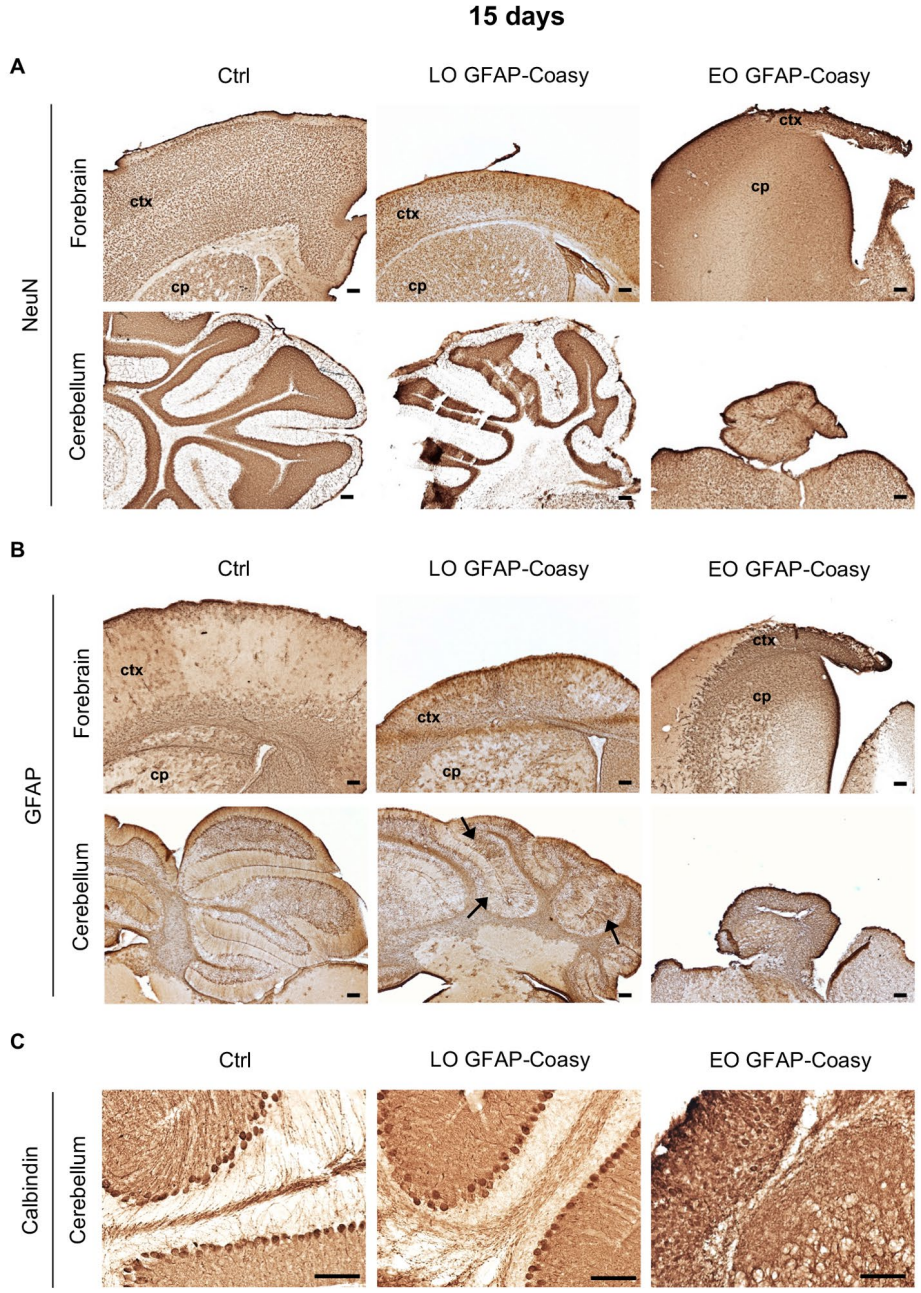
Pathological characterization of LO and EO GFAP-Coasy mice at 15 days. **(A)** Immunohistochemical analysis of coronal forebrain and cerebellum sections performed with NeuN antibody showing alteration in cortical and cerebellar organization in LO GFAP-Coasy mice, and almost total absence of cortex and cerebellum in EO GFAP-Coasy mice. **(B)** Staining with GFAP antibody revealed cortical astrocytosis in both EO and LO GFAP-Coasy animals, and Bregmann glia alteration in LO GFAP-Coasy mice (arrows). **(C)** Detail of cerebellum labeled with calbindin antibody showing slight alteration of Purkinje cells in LO GFAP-Coasy, and their complete disorganization in EO GFAP-Coasy mice. LO, late onset; EO, early onset; ctx, cortex; cp, caudate putamen. Scale bar: 100 μm.

Considering the EO and LO phenotypes, we decided to perform all histological examinations of the brains of both EO and LO GFAP-Coasy mice at two time points: at P15, when the EO GFAP-Coasy mice were compassionately euthanized, and after 6 months when only the LO GFAP-Coasy mice were still alive. At P15, the cerebral cortex of the control animals showed a uniform distribution of NeuN^+^ neurons in all layers. On the contrary, the brains of LO GFAP-Coasy mice revealed a thinning and a disorganization of the cerebral cortex layers, as showed by NeuN staining (Figure 2A). The same staining showed the rarity of NeuN^+^ neurons and the complete absence of the cortex layers in the EO GFAP-Coasy mice. Laminar organization of the cerebellum was also impaired in the LO GFAP-Coasy mice as compared to control animals (Figure 2A). In the EO GFAP-Coasy mice, the cerebellum had not developed properly and only a sketch of the cerebellum was present (Figure 2A).

Furthermore, staining of the same areas with specific antibody against glial fibrillary acidic protein (GFAP), the upregulation of which is a recognized feature of astrogliosis, revealed the presence of astrocytes hyperproliferation, which was more evident in EO as compared to LO GFAP-Coasy mice at P15, and was more prominent in the underdeveloped cortex of EO animals (Figure 2B). Moreover, the organization of Bergmann glia, such as unipolar astrocytes derived from radial glia that reside in the cerebellar ventricular zone and serve as a scaffold for granule cell migration and correct Purkinje cells positioning, appear altered in the LO GFAP-Coasy animals and completely loss in EO GFAP-Coasy mice (Figure 2B).

We then analyzed cerebellar sections of controls, LO, and EO GFAP-Coasy mice at P15 by examining the expression of calbindin, a widely used histochemical marker for Purkinje neurons in the cerebellum. We found that the Purkinje cell layer and molecular layer of LO GFAP-Coasy mice were less defined and disorganized compared to control mice (Figure 2C). The cytoarchitecture of the cerebellum of EO GFAP-Coasy mice was completely disrupted and it was almost impossible to recognize the different layers and identify calbindin^+^ Purkinje cells.

**Figure 3:**
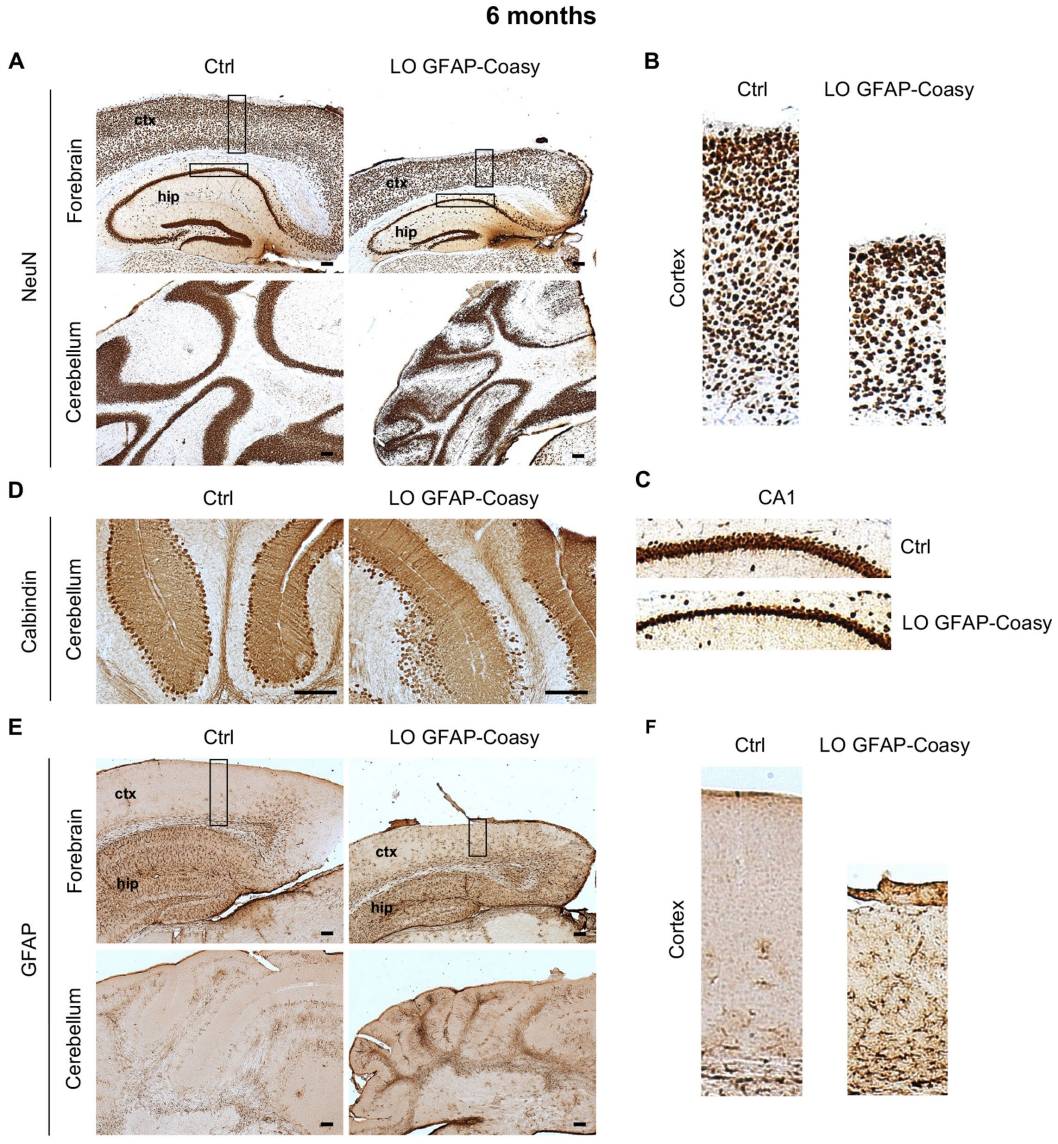
Pathological characterization of LO GFAP-Coasy mice at 6 months. **(A)** Immunohistochemical analysis of coronal forebrain and cerebellum sections performed with NeuN antibody showing severe layer reduction and disorganization of cortex, hippocampus, and granular cerebellar cells. Magnified insets of **(B)** cortex, and **(C)** hippocampal CA1 region are showed. **(D)** Detail of cerebellum labeled with calbindin antibody displaying Purkinje cells loss and disorganization in GFAP-Coasy mice. **(E)** GFAP staining showed high astrocytes hyperproliferation and Bergmann glia alteration in the cortex and cerebellum of GFAP-Coasy animals. **(F)** Magnified insets of cortex are shown. LO, late onset; EO, early onset; ctx, cortex; hip, hippocampus. Scale bar: 100 μm.

Next, we investigate cerebral architecture in the cortex and hippocampus of control and LO GFAP-Coasy mice at 6 months of age. Evaluation of NeuN^+^ neurons in the cortex revealed that LO GFAP-Coasy mice exhibited severe layer reduction and disorganization as shown by histochemical analysis (Figures 3A,B). In addition, NeuN^+^ cells in the CA1 region of the hippocampus showed a reduction in thickness in GFAP-Coasy mice compared to controls (Figure 3C). Analysis of the cerebellum, using calbindin histochemical evaluation, demonstrated that the Purkinje cell layer was disorganized and there was a loss of Purkinje cells (Figure 3D). Finally, histochemical staining of the cortex and cerebellum with GFAP showed high astrocytes hyperproliferation and Bergmann glia alteration in LO GFAP-Coasy mice compared to control mice (Figures 3E,F).

Altogether, these data suggest that the specific ablation of Coasy in astroglial lineage leads to a motor dysfunction which, together with the disorganization of the cerebral cortex and cerebellum, is likely the result of altered radial neuronal migration during neurodevelopment. In addition, a pronounced astrogliosis was observed in the cerebral cortex and cerebellum of GFAP-Coasy mice, which could be the cause or the consequence of aberrant radial neuronal migration.

### Molecular and biochemical characterization of EO and LO GFAP-Coasy animals

**Figure 4:**
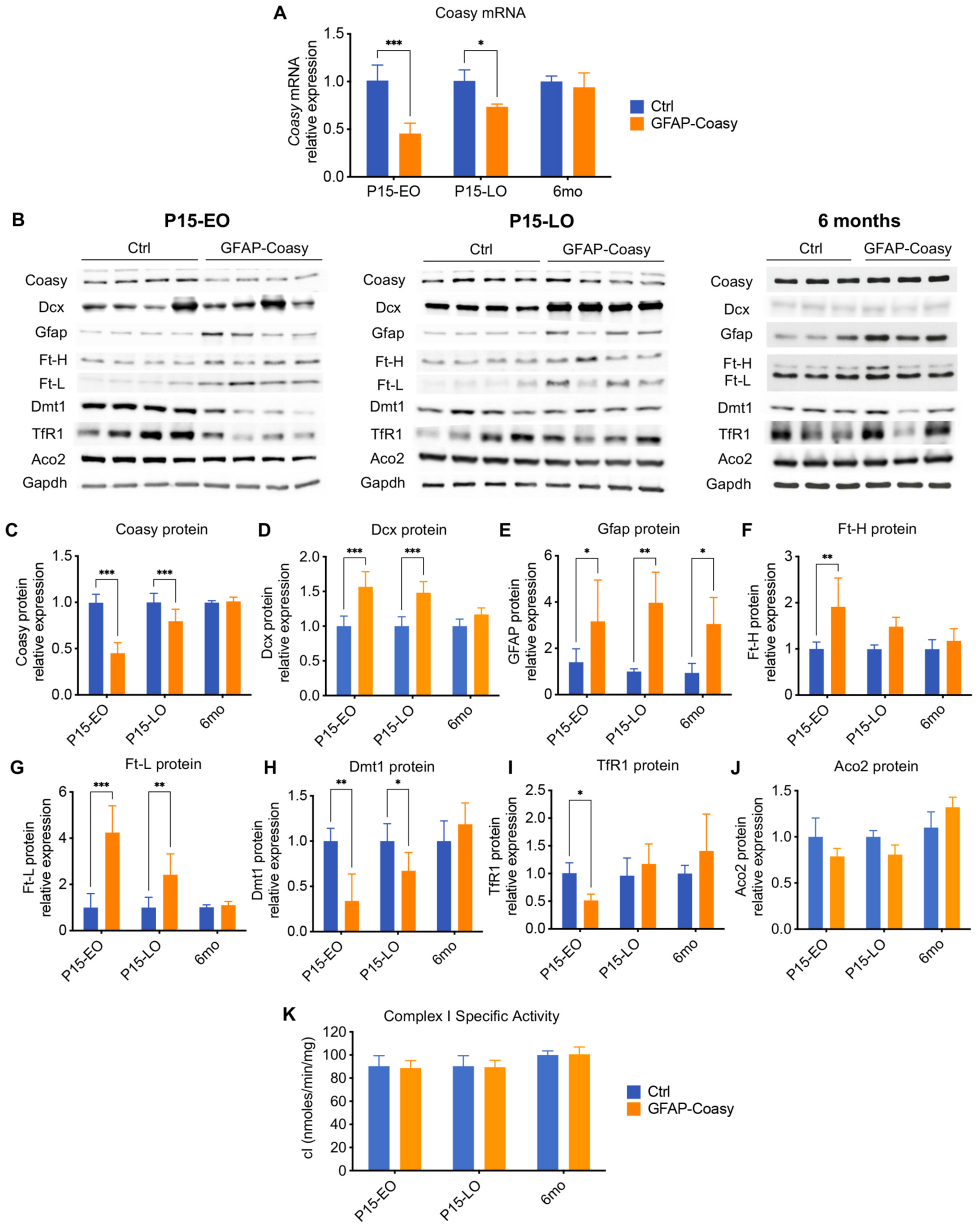
Molecular and biochemical characterization of GFAP-Coasy mice. **(A)** Relative Coasy mRNA expression at 15 days and 6 months in Ctrl (n = 4) and GFAP-Coasy (n = 4) mice brain, tested by qPCR. **(B)** Representative images of Western blot analysis and **(C–J)** densitometric quantification of **(C)** Coasy, **(D)** Dcx, **(E)** Gfap, **(F)** Ft-H, **(G)** Ft-L, **(H)** Dmt1, **(I)** TfR1, and **(J)** Aco2 at 15 days and 6 months in Ctrl and GFAP-Coasy mice brain. **(K)** Specific activity of mitochondrial respiratory chain complex I, expressed as nmoles/min/mg of protein. *p < 0.05, ** *p* < 0.01; ***p < 0.001 (two-way ANOVA).

To assess the level of Coasy transcript in the cortex of control, EO, and LO GFAP-Coasy mice, we measured relative Coasy mRNA expression at P15 and 6 months. We found that Coasy expression significantly decreased in EO and to a lesser extent in LO GFAP-Coasy mice at P15, whereas expression at 6 months was comparable between control and LO GFAP-Coasy mice (Figure 4A). We then measured Coasy protein levels in the same mice by Western blot analysis and showed that the Coasy protein recapitulates mRNA expression levels, being significantly reduced at P15 in EO and LO GFAP-Coasy mice compared to controls, while this difference disappears at 6 months when Coasy protein levels are comparable between control and LO GFAP-Coasy mice (Figures 4B,C).

As histological analysis suggested neurodevelopmental defects in EO and LO GFAP-Coasy mice, we performed quantification of doublecortin (Dcx) protein, which is a marker for neuronal precursors or immature neurons. Immunoblot analysis showed significant accumulation of Dcx in the EO and LO GFAP-Coasy brain (Figures 4B,D), indicating impaired neuronal differentiation and maturation.

Considering the astrogliosis observed in the cortex of EO and LO GFAP-Coasy mice, we also examined the amount of the Gfap protein by Western blot analysis and found that Gfap protein levels were significantly increased in both EO and LO GFAP-Coasy mice at P15 and 6 months of age (Figures 4B,E).

Since the disorders associated with *Coasy* may be related to iron metabolism, we also analyzed by Western blot the levels of proteins involved in the control of cellular iron homeostasis, such as: Ferritin Light (Ft-L) and Heavy (Ft-H) chains, which together form the main complex that stores iron in the cytoplasm of the cell; the transferrin receptor 1 (TfR1), which imports iron into the cell by internalizing the transferrin-iron complex through receptor-mediated endocytosis; the divalent metal transporter 1 (Dmt1), which pumps iron from the endosome into the cytosol (Figure 4B). Densitometric quantification of the results showed that Ft-H protein is significantly increased in EO GFAP-Coasy mice at day 15, whereas no differences were observed in LO GFAP-Coasy mice at P15 and month 6 (Figure 4F). In addition, quantification of Ft-L showed that this protein is significantly increased in EO and LO GFAP-Coasy mice at P15, whereas this difference disappears at month 6 (Figure 4G). Similarly, Dmt1 protein is significantly reduced in both EO and LO GFAP-Coasy mice at P15 (Figure 4H), and TfR1 levels are decreased only in EO but not in LO GFAP-Coasy mice (Figure 4I). These differences in either Dmt1 or TfR1 also disappeared at 6 months (Figures 4H,I). The levels of Ft-L, Ft-H, Dmt1, and TfR1 proteins are tightly regulated by the iron-regulating proteins IRP1 and IRP2. When intracellular iron is low, the IRPs bind to the mRNAs of TfR1 and Dmt1, preventing their degradation and enabling increased iron import. At the same time, IRPs bind to the mRNAs of ferritins, inhibiting their synthesis. Under iron-rich conditions, IRP1 becomes aconitase, and IRP2 is degraded, leading to more iron storage and less iron import (Hentze et al., 2010). Interestingly, the protein levels of Ft-L and Dmt1 reflect to some extent the level of the Coasy protein: when Coasy is reduced, Ft-L and Dmt1 are altered. This is also true for Ft-H and TfR1, but only in EO GFAP-Coasy mice at P15.

In the cell, iron is mainly used to form iron–sulfur clusters (ISCs), which are essential for the proper functioning of numerous enzymes involved in ATP generation, the Krebs cycle, or DNA synthesis, maintenance, and repair (Rouault, 2012). Therefore, we analyzed the protein content of one of the major ISC-containing proteins, such as aconitase 2 (Aco2), as well as the activity of the ISC-containing mitochondrial respiratory chain complex I (cI), in total brain homogenates. We did not detect a significant change in either Aco2 protein levels (Figures 4B,J), or in cI specific activity (Figure 4K).

### Characterization of primary astrocytes derived from GFAP-Coasy mice

**Figure 5:**
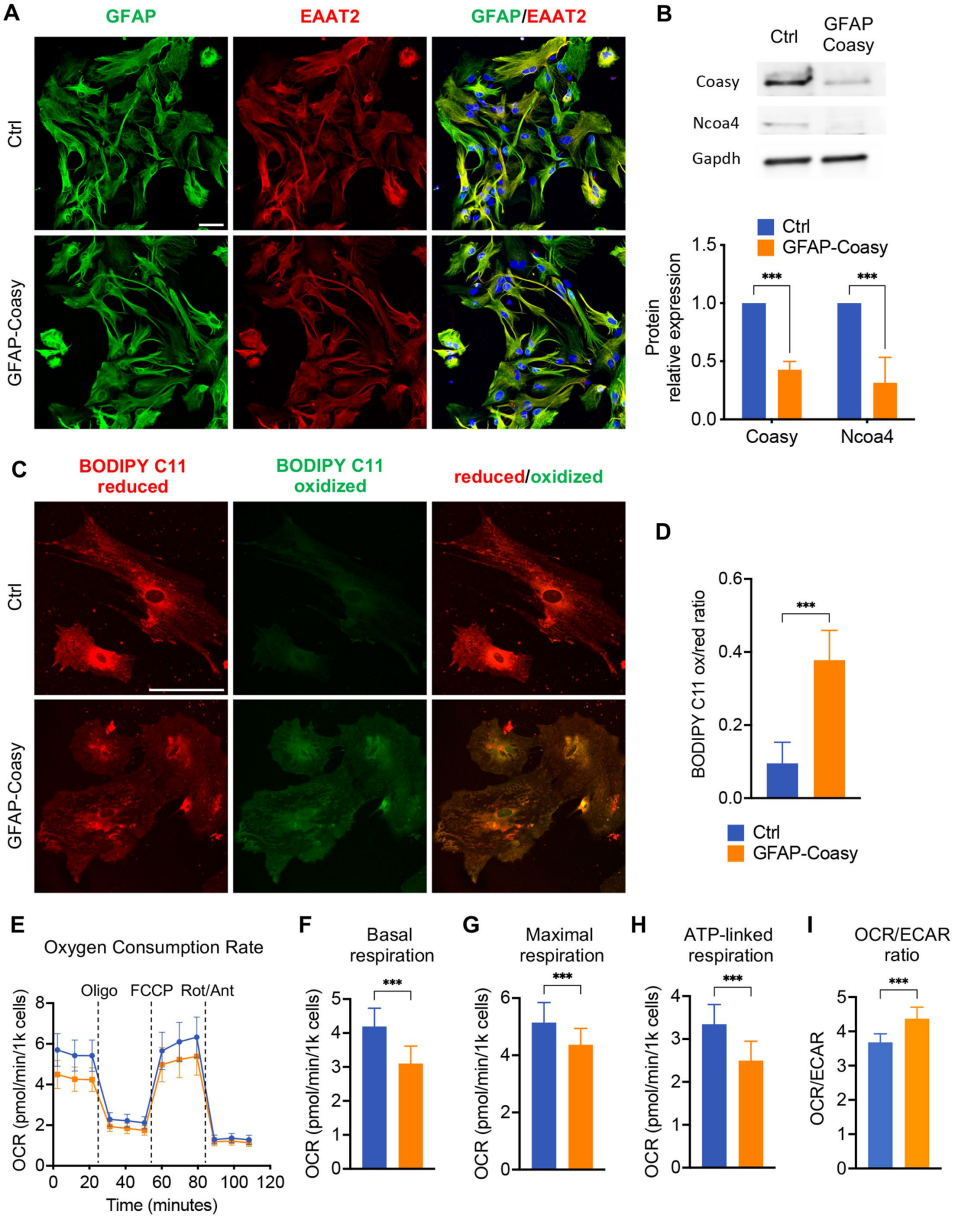
Analysis of GFAP-Coasy primary astrocytes. **(A)** Representative confocal immunofluorescence images of astrocytes isolated from Ctrl and GFAP-Coasy hippocampus. Astrocytes were stained with the specific markers glial fibrillary acidic protein (GFAP) and excitatory amino acid transporter 2 (EAAT2). Nuclei were stained with Hoechst. Scale bar: 50100 μm. **(B)** Representative images of Western blot analysis and densitometric quantification of Coasy and Ncoa4 in homogenates from primary astrocytes derived from Ctrl and GFAP-Coasy animals. ***p < 0.001 (two-way ANOVA). **(C)** Representative confocal images of lipid peroxidation in Ctrl and GFAP-Coasy primary astrocytes by BODIPY C11 staining. Scale bar: 50100 μm. **(D)** Fluorescence quantification of BODIPY C11 staining, calculated by the ratio between oxidized and reduced signals on Ctrl (*n* = 10) and GFAP-Coasy (*n* = 10) images, showing elevated lipid peroxidation in GFAP-Coasy compared with control cells. ***p < 0.001 (Student’s *t*-test). **(E)** Oxygen consumption rate (OCR) of astrocytes, expressed as pmoles O_2_/min/1000 cells, under basal conditions and after injection of oligomycin (Oligo), carbonyl cyanide 4-(trifluoromethoxy) phenylhydrazone (FCCP), rotenone (Rot) and antimycin A (Ant), from Ctrl (*n* = 22) and GFAP-Coasy (*n* = 22) cells. **(F)** Maximal, **(G)** basal, and **(H)** ATP-linked respirations were calculated from OCR traces, showing mitochondrial energy impairment in GFAP-Coasy primary astrocytes compared with Ctrl. **(I)** OCR on ECAR ratio calculated at basal points. ***p < 0.001 (Student’s *t*-test).

GFAP-Coasy mice were generated by specific ablation of the Coasy gene in the astroglial lineage. Nevertheless, all histochemical, biochemical, and molecular analyses, were performed on the cerebral cortex and cerebellum of these mice. To specifically investigate the consequences of Coasy ablation in astrocytes, we isolated these cells from GFAP-Coasy mice and control littermates’ hippocampi at P3. First, we characterized these cells by immunofluorescence analysis with antibodies specific for astrocytes, such as GFAP and excitatory amino acid transporter 2 (EAAT2). This analysis revealed the presence of cells that reacted positively to both antibodies, confirming their specific astrocytic identity (Figure 5A). We then performed Western blot analysis to evaluate the expression levels of the Coasy protein. These analyses showed that Coasy was significantly decreased in GFAP-Coasy-derived astrocytes compared to control cells (Figure 5B).

Since we found increased levels of Ft-L and Ft-H in the cortex of GFAP-Coasy mice, we tested the levels of the nuclear receptor coactivator 4 (NCOA4), the cargo which mediates the cytoplasmic iron release by the autophagy ferritin degradation process called ferritinophagy (Mancias et al., 2014). Ncoa4 levels were reduced in GFAP-Coasy astrocytes (Figure 5B), which is typical of an iron overload scenario (Bellelli et al., 2016; Santambrogio et al., 2022).

To further investigate the consequences of Coasy ablation in astrocytes, we examined the presence of increased oxidative stress using BODIPY C11, a fluorescent probe for lipid peroxidation that shifts its fluorescence emission from red to green under oxidative conditions. As highlighted in Figure 5C, green fluorescence was virtually absent in control astrocytes, whereas we observed an increase in this fluorescence in GFAP-Coasy astrocytes. By quantitatively evaluating the ratio between oxidized and reduced BODIPY C11, we could show that this ratio was significantly higher in GFAP-Coasy astrocytes compared to control astrocytes (Figure 5D).

As mitochondria are the main subcellular storage and utilization site for CoA and acyl-CoAs, we investigated mitochondrial respiration using microscale oxygraphy, which provides a real-time measurement of oxygen consumption rate (OCR), a direct indicator of mitochondrial energetic function, and the extracellular acidification rate (ECAR), which is proportional to the production of lactic acid, and thus reflects the rate of glucose utilization via anaerobic glycolysis under basal conditions. GFAP-Coasy astrocytes were characterized by an overall impaired mitochondrial respiration (Figure 5E), and showed a significant decrease in basal (Figure 5F), maximal (Figure 5G), and ATP-linked (Figure 5H) respiration. In addition, the lower OCR/ECAR ratio in GFAP-Coasy cells indicates a relatively higher dependence on glycolysis compared to Ctrl (Figure 5I).

These data suggest that ablation of Coasy in astrocytes is associated with increased oxidative stress and impaired mitochondrial functionality, which could have a direct negative effect on GFAP-Coasy astrocytes but could also be detrimental to normal brain development and health in the context of the whole brain.

## Discussion

Genetic variants of the *COASY* gene are responsible for a broad spectrum of clinical phenotypes, ranging from an invariable lethal phenotype with prenatal onset pontocerebellar hypoplasia, microcephaly, and arthrogryposis (PCH12), to neurological and neurodegenerative diseases characterized by heterogeneous symptoms and variable onset in childhood and adulthood (CoPAN).

To investigate the pathogenic mechanisms of these diseases, we previously generated a mouse model in which the *Coasy* gene was specifically deleted in neurons by the action of a Cre recombinase under the control of the human synapsin 1 promoter (Syn1-Coasy) (Di Meo et al., 2020). These Syn1-Coasy mice exhibited a severe neurological phenotype associated with early death.

To further complement this model and in an attempt to clarify the origin of the clinical heterogeneity observed in patients, also considering that the constitutive *Coasy* knock-out is embryonically lethal, we took advantage of the Coasy^flox^ mice, by crossing these mice with a mouse line expressing the Cre recombinase under the control of the human GFAP promoter (Zhuo et al., 2001).

We observed that the GFAP-Coasy mouse model, obtained by the specific ablation of the *Coasy* gene in radial glia during brain development and its descendants, such as astrocytes and some cortical neurons, exhibited a neurodevelopmental disorder characterized by the disorganization of the cerebral cortex and cerebellar layers with variable severity of the clinical phenotypes associated with early death or normal life expectancy. Interestingly, we showed that EO GFAP-Coasy mice had a severe clinical phenotype characterized by death at P15, a severe reduction in the thickness of the cerebral cortex, and the almost complete absence of the cerebellum. However, we also observed a distinct milder phenotype in LO GFAP-Coasy mice, which does not affect the animals’ life expectancy but is associated with disorganization of the cerebral cortex and cerebellum layers, and severe movement disorders. At the molecular level, the two contrasting phenotypes seem to correlate with a significant variation of the Coasy protein in the brain: in both EO and LO GFAP-Coasy mice, we observed a significant reduction of the Coasy protein at P15 compared to control mice, whereas, in LO GFAP-Coasy mice, Coasy protein levels at 6 months were comparable to those of control mice. This variability could be due to epigenetic modifications, which may prevent Cre recombinase from removing the loxP-flanked *Coasy* gene. However, it has been reported that Cre recombinase transgenes can have different expression strengths, which may determine the heterogeneity of Cre-mediated recombination in different animals (Requardt et al., 2009). This possibility could explain the presence of the Coasy protein at 6 months but does not justify why in the presence of a normal level of Coasy protein, the mice manifested neurological phenotypes that correlate with histological alteration of the brains and cerebellum. One hypothesis could be that the ablation of *Coasy* in the radial glia induces the degeneration of these cells during the late, post-natal neurodevelopment, irreversibly impairing cortical and cerebellar organization. Thus, only Cre-resistant cells survive, making up what remains of the adult cortex and cerebellum. It has been shown that GFAP-Cre transgene is not only active in astrocytes, but also in radial glia during development, so that recombination could take place in neurons (Garcia et al., 2004). This dual expression could explain the very severe phenotype of EO GFAP-Coasy mice, characterized by neurodevelopmental defects, as well as by the marked reduction of the Coasy protein. All of the above events could likely contribute to generate the phenotypic variability observed in GFAP-Coasy mice.

To understand what is the consequence of *Coasy* ablation in astrocytes, we harvested these cells from the brain of P3 GFAP-Coasy mice and analyzed them *in vitro*. We found that lipid peroxidation was significantly increased in GFAP-Coasy-derived astrocytes, which may be related to iron dyshomeostasis. Accordingly, we recently showed that human astrocytes differentiated from iPS cells derived from individuals carrying variants in the *PANK2* gene showed marked lipid and protein peroxidation due to massive iron accumulation, leading to an iron-dependent, lipid-peroxidation-driven cell death cascade known as ferroptosis (Santambrogio et al., 2022). We also demonstrated that GFAP-Coasy-derived astrocytes displayed alterations in cellular energetic functionality, showing lower mitochondrial respiration in favor of higher glycolytic dependence. It is important to consider that during neurogenesis there is a controlled balance between glycolysis and oxidative phosphorylation (OXPHOS). While self-renewing cells, such as radial glia, show higher glycolysis with increased lactate production and lower OXPHOS, differentiated neural progenitor cells show lower glycolysis and higher OXPHOS (Namba et al., 2021). Consistent with these findings, the alteration of the balance between glycolysis and OXPHOS in mouse embryonic development has been associated with impaired cortical neurogenesis (Khacho et al., 2017). Therefore, our results may also indicate poor differentiation and increased proliferation of progenitor cells that are more dependent on glycolysis for energy production. These data are further confirmed by the increased expression of the protein doublecortin (Dcx), a marker for neuronal progenitor cells or immature neurons, indicating impaired neuronal differentiation and maturation.

The disorders associated with Coasy are also linked to impaired iron metabolism, and we clearly demonstrated that iron metabolism is significantly de-regulated in the absence of the Coasy protein. Overall, our data suggest a link between ablation of *Coasy* and abnormal iron metabolism, which is an important regulator of neurodifferentiation.

Despite some limitations of our GFAP-Coasy mouse model, we were able to clearly link the absence of the *Coasy* gene in astroglial lineage, to a neurodevelopmental disorder, that had previously only been observed in a *Coasy* knock-out zebrafish model (Khatri et al., 2016) and in patients with PCH12 (van Dijk et al., 2018; Mishra et al., 2022). When we ablated the *Coasy* gene in neurons (Di Meo et al., 2020), the phenotype of the mice was characterized by early death at day 15, but was not accompanied by neuronal loss or disorganization of the cerebral cortex or cerebellar layers. In addition, the GFAP-Coasy mouse model showed increased astrogliosis both in the cerebral cortex and cerebellum, which could be related to neuronal cell death, as we did not observe these reactive astrocytes in the Syn1-Coasy mice, which had movement disorders and disturbed iron metabolism in common with GFAP-Coasy mice. Considering the results obtained in Syn1- and GFAP-Coasy mice, we hypothesized that the removal of Coasy gene in different cell types (neurons and astrocytes in our models), may lead to heterogeneous phenotypes, depending on the expression level of the Coasy protein in specific cell types of the brain, which could then modulate CoA availability. It can therefore be speculated that, depending on the type of mutation affecting the amount of Coasy protein, it may have a variable effect in the different cell types of our body and could possibly explain the heterogeneity of the clinical presentations.

Our study also emphasizes and supports evidence suggesting that proteins involved in neurodegenerative diseases may also play an important role in brain development. This dual role is clearly defined for proteins associated with common neurodegenerative diseases such as Alzheimer’s, Frontotemporal dementia, Huntington’s disease, and Parkinson’s disease (Schor and Bianchi, 2021). Based on our experimental observations *in vitro*, *in vivo*, and in patients, we would like to propose that the COASY enzyme, which is involved in the synthesis of CoA, a vital molecule, can be included in the list of genes playing a crucial role during neurodevelopment and in the neurodegenerative process.

Given the crucial role of the COASY enzyme and the complexity behind the regulation of its expression and, consequently CoA levels, it is not surprising that *COASY* variants are also responsible for other clinical phenotypes such as riboflavin-responsive lipid storage myopathy (Zheng et al., 2024). Although the pathological mechanisms by which *COASY* variants cause a broad spectrum of clinical phenotypes are not clarified, it is possible to speculate that fine-tuning of CoA levels, probably in different subcellular localizations, may represent another factor contributing to the heterogeneity of the clinical presentations.

## Data Availability

The raw data supporting the conclusions of this article will be made available by the authors, without undue reservation.

## Supplementary material

SUPPLEMENTARY VIDEO S1 Video showing the severe phenotype displayed by two EO GFAP-Coasy mice at P15.
